# The Tuning of Strain in Layered Structure Oxide Cathodes for Lithium-Ion Batteries

**DOI:** 10.34133/research.0489

**Published:** 2024-09-18

**Authors:** Xianji Qiao, Liguang Wang, Jun Lu

**Affiliations:** College of Chemical and Biological Engineering, Zhejiang University, Hangzhou 310058, China.

## Abstract

Layered structure oxides have emerged as highly promising cathode materials for lithium-ion batteries. In these cathode materials, volume variation related to anisotropic lattice strain during Li^+^ insertion/extraction, however, can induce critical structural instability and electrochemical degradation upon cycling. Despite extensive research efforts, solving the issues of lattice strain and mechanical fatigue remains a challenge. This perspective aims to establish the “structure–property relationship” between the degradation mechanism of the layered oxide cathode due to lattice strain and the structural evolution during cycling. By addressing these issues, we aim to guide the improvement of electrochemical performance, thereby facilitating the widespread adoption of these materials in future high-energy density lithium-ion batteries.

To date, most of the reported layered oxide cathodes suffer from severe lattice strain issues, resulting in crystal structure fatigue and particle microcracks [1,2]. Fundamentally, lattice strain is unavoidable in insertion layered oxides due to Li^+^ insertion/extraction, stemming from the changes in ion size of redox-active elements and electrostatic repulsions of oxygen. The changes in ion size induces a contraction of transition metal–oxygen octahedrons (TMO_6_) such as Li(Ni_1−*x*−*y*_Co*_x_*Mn*_y_*)O_2_ (NCM) and Li-rich layered-oxide cathodes, which leads to the reduction of the lattice parameter *a*(*b*) throughout the whole charging process. Meanwhile, the change in electrostatic interaction that follows (primarily arising from the electrostatic interaction between oxygen–oxygen) has a substantial effect on the parameter *c*, severely pronounced in the high-voltage region where the *c*-spacing shows rapid contraction [1] for NCM. However, the change of the *c* lattice parameter is less severe for Li-rich layered-oxide cathodes compared to NCM due to the oxygen ions’ charge compensation [2]. Furthermore, the inhomogeneous reaction due to the inherent reaction sequence induces lattice distortion inside the primary particle crystals [3], leading to lattice strain and irreversible phase transition, and is recognized as the origin of the deterioration of layered oxides [1,2]. Although the undesired lattice displacements are often considered as the root cause of the structural instability, the structural and chemical heterogeneity at the mesoscale further complicates the system [4].

Extensive efforts have focused on suppressing the structural degradations arising from lattice variation using various strategies, such as (a) elemental doping, (b) microstructure engineering, and (c) single-crystal development [5]. Nonetheless, these traditional strategies are unable to alleviate the inherent degradation stemming from the evolution of anisotropic lattice strain in layered oxides [1,2]. Thus, searching for effective strategy to achieve a high degree of control of the rising lattice strain is crucial for enhancing structural stability. The inherent reaction sequence inside the primary crystals due to electron and lithium ion transfer (Figure A) [3] and long-term cycling leads to an inhomogeneous chemical state across the particle [6]. This inhomogeneity results in localized crystal structure variation, causing lattice strain inside the particle. Enhancing the cation disorder in layered oxide cathodes has demonstrated the ability to achieve high structural stability, as it facilitates homogeneous strain distribution during (de)lithiation [7,8]. Consequently, an effective strategy for developing low-strain layered structure oxides involves enhancing the degree of cation disorder. For instance, Ni-rich layered oxides (Li_1–m_(Ni_0.94_Al_0.06_)_1+m_O_2_) with higher cation disorder demonstrate a stable crystal structure with reduced lattice parameter variation and homogenous reactivity, leading to a dramatic decrease in interfacial strain, which is a key contributor to the capacity decay. Furthermore, this strategy can be extended to high-entropy structure design as an effective approach to resist against lattice strain due to a larger energy increase with the lattice contraction and expansion benefitting from the cation disorder (Figure B) [9]. On the other hand, the migration of transition metal (TM) ions also plays a pivotal role in strain evolution. Therefore, the tuning of TM migration is helpful to construct low-strain layered oxide cathodes. For instance, designing a new type of O2 lattice stacking sequence (Li*_x_*(Li_0.2_Ni_0.2_Mn_0.6_)O_2_, where *x* ≈ 0.83) through regulating the oxygen lattice of lithium-rich layered oxides to enhance the reversibility of TM migration significantly suppresses the lattice distortion and defects in contrast with those of the O3 type, thereby mitigating lattice strain (Figure C) [10]. Recently, a new strategy was proposed by incorporating a strain retardant into the bulk phase to form a low-strain coherent structure, involving a robust matrix capable of sustaining the mechanochemical stability due to the high-threshold energy barriers for phase transitions in the electrochemically inactive strain-retardant phase (Figure D) [1]. Based on this strategy, research on using alternative inherent high stability phases such as spinel-like structures [11] also gained great attention as effective approaches in solving lattice strain, which open a new avenue for cathode development.

**Figure.**
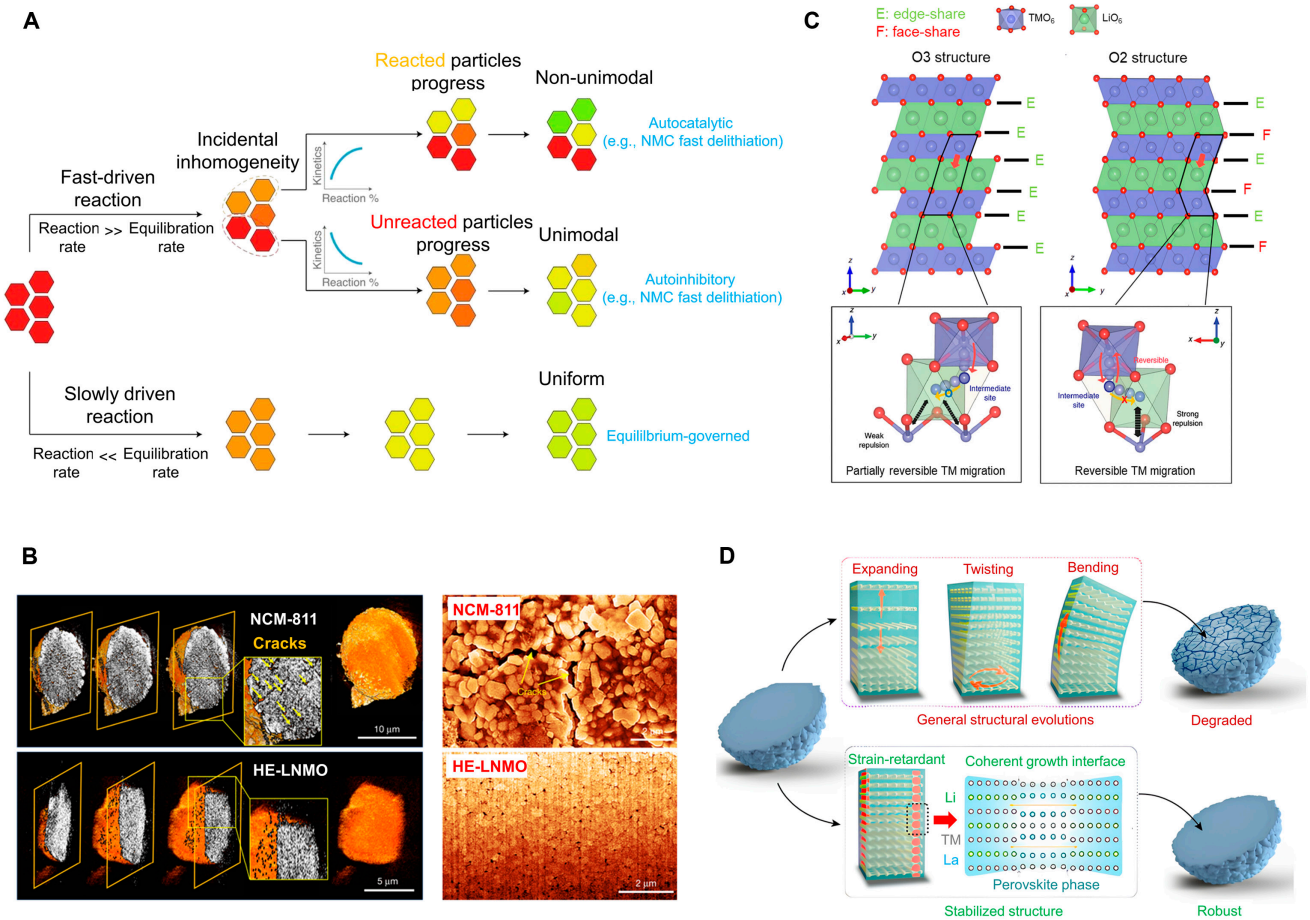
Schematic diagram of (A) inhomogeneous reactions in electrochemically driven systems; (B) synchrotron-based TXM tomography showing the 3D structure of the NMC-811 and high-entropy structure HE-LNMO secondary particles after long-term cycling; (C) crystal structures of O3-type and O2-type lithium layered oxides; and (D) material structural design with a strain retardant.

In summary, effective strategies for degradation caused by the lattice strain remain unresolved so far, although previous research efforts have sought diverse solutions. Strategies concerning the structure design to regulate the rising lattice strain include enhancing the cation disorder, modifying the TM migration path, and introducing a strain retardant. These strategies offer new perspectives on clarifying the stability mechanism of layered oxide cathodes and developing high-energy-density batteries for practical use with feasible approaches.
